# Impact of Thromboprophylaxis across the US Acute Care Setting

**DOI:** 10.1371/journal.pone.0121429

**Published:** 2015-03-27

**Authors:** Wei Huang, Frederick A. Anderson, Sophie K. Rushton-Smith, Alexander T. Cohen

**Affiliations:** 1 Center for Outcomes Research, University of Massachusetts Medical School, Worcester, Massachusetts, United States of America; 2 Department of Haematological Medicine, Guys and St Thomas' NHS Foundation Trust, King’s College, London, United Kingdom; Maastricht University Medical Center, NETHERLANDS

## Abstract

**Background:**

The risk of venous thromboembolism (VTE) can be reduced by appropriate use of anticoagulant prophylaxis. VTE prophylaxis does, however, remain substantially underused, particularly among acutely ill medical inpatients. We sought to evaluate the clinical and economic impact of increasing use of American College of Chest Physicians (ACCP)-recommended VTE prophylaxis among medical inpatients from a US healthcare system perspective.

**Methods and Findings:**

In this retrospective database cost-effectiveness evaluation, a decision-tree model was developed to estimate deaths within 30 days of admission and outcomes attributable to VTE that might have been averted by use of low-molecular-weight heparin (LMWH) or unfractionated heparin (UFH). Incremental cost-effectiveness ratio was calculated using “no prophylaxis” as the comparator. Data from the ENDORSE US medical inpatients and the US nationwide Inpatient Sample (NIS) were used to estimate the annual number of eligible inpatients who failed to receive ACCP-recommended VTE prophylaxis. The cost-effectiveness analysis indicated that VTE-prevention strategies would reduce deaths by 0.5% and 0.3%, comparing LMWH and UFH strategies with no prophylaxis, translating into savings of $50,637 and $25,714, respectively, per death averted. The ENDORSE findings indicated that 51.1% of US medical inpatients were at ACCP-defined VTE risk, 47.5% of whom received ACCP-recommended prophylaxis. By extrapolating these findings to the NIS and applying cost-effectives analysis results, the full implementation of ACCP guidelines would reduce number of deaths (by 15,875 if using LMWH or 10,201 if using UFH), and was extrapolated to calculate the cost reduction of $803M for LMWH and $262M for UFH.

**Conclusions:**

Efforts to improve VTE prophylaxis use in acutely ill inpatients are warranted due to the potential for reducing VTE-attributable deaths, with net cost savings to healthcare systems.

## Introduction

Venous thromboembolism (VTE), comprising the conditions of deep vein thrombosis (DVT) and pulmonary embolism (PE), is associated with considerable long-term morbidity, functional disability and mortality [1]. VTE is estimated to be the third most common cardiovascular disorder, after the acute coronary syndromes and ischemic stroke [2]. Hospitalized patients are at particularly high risk for developing VTE [1], with 5–10% of all deaths among hospitalized patients associated with PE [3, 4]. Non-surgical patients account for 70–80% of all fatal and 50–70% of symptomatic thromboembolic events [4–7]. In addition to the clinical impact of VTE, the economic burden of this disease is considerable [8, 9]. Direct medical costs for the treatment of patients with nonfatal VTE are estimated to be between 6 and 8 billion dollars based on 2004 provider payments in the United States (US) [9, 10].

Numerous randomized trials have demonstrated that the risk of VTE can be reduced in patients who receive anticoagulant prophylaxis [6, 7]. Despite evidence-based guidelines recommending prophylaxis for at-risk patients [6, 7], the Epidemiologic International Day for the Evaluation of Patients at Risk for Venous Thromboembolism in the Acute Hospital Care Setting (ENDORSE) study found that VTE prophylaxis remains substantially underused, particularly among acutely ill medical inpatients [11]. Presumptive reasons for this underuse include concerns related to the cost of prophylaxis, low patient compliance with self-injectable out-of-hospital medications, and physician concerns over possible bleeding complications or development of heparin-induced thrombocytopenia (HIT), particularly among patients who receive unfractionated heparin (UFH) [12]. The world faces increasing pressure to contain the costs of healthcare; and studies measuring the cost-effectiveness of VTE prevention are becoming increasingly important for gathering data to support evidence-based decisions.

In this analysis, we evaluate the potential economic impact of ongoing efforts to increase physician adherence to American College of Chest Physicians (ACCP)-recommended guidelines on VTE prophylaxis, along with the estimated benefits of reducing VTE-attributable events, among hospitalized acutely ill medical patients from a US healthcare system perspective. In addition, we evaluate the incremental cost-effectiveness ratios (ICERs) of the additional cost for ACCP-recommended VTE prophylaxis per episode of VTE-attributable death averted (versus no prophylaxis) among medical inpatients at ACCP-defined moderate or high VTE risk in the US acute care hospital setting.

## Methods

### Ethics

The ENDORSE study was approved by the institutional review committee at UMass Medical School. Signed patient consent was required in Brazil, Greece, and Hungary. Patient information was anonymized and de-identified prior to analysis.

### Study Design

The ENDORSE study was an observational, cross-sectional study conducted between August 2006 and January 2007. A total of 358 hospitals were selected at random from authoritative lists of acute care hospitals (American Hospital Association List for US) in 32 participating countries. The study was designed to assess the number of patients at moderate or high risk for VTE in the acute care hospital setting and to determine the proportion of these at-risk patients who received prophylaxis as recommended by the (then current) 2004 ACCP evidence-based consensus guidelines [6]. Patient data were collected from a review of hospital charts and recorded in case report forms by trained data abstractors. The full methods for the ENDORSE study have been published [11]. In this analysis, we focused on the ENDORSE US medical inpatients, combined with publically available US data from the Nationwide Inpatient Sample (NIS) [13], to estimate the total annual number of medical inpatients at ACCP-defined VTE risk and the proportion of at-risk patients who fail to receive ACCP-recommended VTE prophylaxis. The NIS data is the largest all-payer inpatient care database that is publicly available in the US, containing data from approximately 8 million hospital stays in about 1000 hospitals sampled to approximate a 20% stratified sample of US community hospitals, and weighted to produce national estimates [13]. Inpatient stay records in the NIS include clinical and resource use information typically available from discharge abstracts [13]. The proportion of patients at clinically recognized bleeding risk (i.e. contraindication to anticoagulation) was estimated based on a previous study [14].

To match the ENDORSE study criteria, the total annual discharges of medical inpatients in the NIS 2006 database was defined as patients who did not go to the operating room, were aged ≥40 years, and had a length of hospital stay ≥2 days. The algorithm developed by Anderson et al [15], according to the primary, secondary, and tertiary discharge diagnostic codes of the ACCP guideline criteria (heart failure, respiratory failure, sepsis, pneumonia, cancer, stroke, acute myocardial infarction, nonsurgical trauma, arthropathy/spondylopathy, paralysis/coma), was used to identify medical inpatients at ACCP-defined VTE risk in the NIS database.

### Decision Analysis Model

A decision-tree model was developed based on a model published by McGarry et al, with updated cost estimates (Table 1 and Table 2; S1 Fig.) [16–51]. This model estimated deaths within 30 days of hospital admission attributable to VTE that might have been averted by the use of ACCP-recommended VTE prophylaxis among a hypothetical cohort of 10,000 acutely ill medical inpatients at ACCP-defined VTE risk. The present comparison of prophylaxis strategies was based on: 1) enoxaparin, a representative low-molecular-weight heparin (LMWH), 40 mg, administered subcutaneously once daily for 7 days; 2) unfractionated heparin (UFH), 5000 IU, administered subcutaneously twice daily for 7 days; and 3) no prophylaxis.

**Table 1:** Probability of incident VTE and AEs within 30 days of hospital admission.

	LMWH (Enoxaparin 40 mg qd)	UFH (5000 IU bid)	None	Ref(s)
Efficacy of prophylaxis				
*P*(DVT)	0.055	0.066	0.142	[18, 19]
Safety of prophylaxis				
*P*(bleed)	0.031	0.058	0.020	[19–22]
*P*(HIT)	0.001	0.010	0.000	[19–21, 23]
Consequences of AEs				
*P*(major bleed|bleed)	0.185	0.185	0.185	[19–22, 24]
*P*(death|major bleed)	0.148	0.148	0.148	[25]
*P*(symptomatic HIT|HIT)	0.543	0.543	0.543	[19, 23, 26–28]
*P*(death|symptomatic HIT)	0.098	0.098	0.098	[29]
Efficacy and safety of DVT treatment				
*P*(bleed|DVT treatment)	0.083	0.083	0.083	[30, 31]
*P*(HIT|DVT treatment)	0.012	0.012	0.012	[30, 31]
*P*(PE|DVT treatment, DVT)	0.018	0.018	0.018	[30, 31]
Efficacy and safety of PE treatment				
*P*(death|PE treatment, +PE)	0.015	0.015	0.015	[32]
*P*(death|PE treatment, −PE)	0.003	0.003	0.003	[33]
Natural history				
*P*(sudden death|PE)	0.100	0.100	0.100	[34, 35]
*P*(PE|no DVT treatment, DVT)	0.511	0.511	0.511	[36, 37]
*P*(death|no PE tx treatment PE)	0.260	0.260	0.260	[38]
*P*(death|underlying illness)	0.100	0.100	0.100	Assumption
Diagnosis of DVT				
*P*(+clinical diagnosis|+DVT)	0.657	0.657	0.657	[39, 40]
*P*(−clinical diagnosis|−DVT)	0.869	0.869	0.869	[39, 40]
*P*(+ultrasound|+DVT)	0.960	0.960	0.960	[41–43]
*P*(−ultrasound|−DVT)	0.962	0.962	0.962	[41–43]
Diagnosis of PE				
*P*(+clinical diagnosis|+PE)	0.291	0.291	0.291	[37, 44–49]
*P*(−clinical diagnosis|−PE)	0.910	0.910	0.910	[44]
*P*(+CT scan|+PE)	0.760	0.760	0.760	[49–51]
*P*(−CT scan|−PE)	0.894	0.894	0.894	[49–51]
*P*(+V/Q scan|+PE)	0.410	0.410	0.410	[44]
*P*(−V/Q scan|−PE)	0.970	0.970	0.970	[44]
*P*(+scan (average)|+PE)	0.585	0.585	0.585	Av. of CT and V/Q scans
*P*(−scan (average)|−PE)	0.932	0.932	0.932	Av. of CT and V/Q scans

AE, adverse event; Av., average; bid, twice daily; CT, computed tomography; DVT, deep vein thrombosis; HIT, heparin-induced thrombocytopenia; IU, international units; LMWH, low-molecular-weight heparin; P, probability; PE, pulmonary embolism; qd, once daily; UFH, unfractionated heparin; VTE, venous thromboembolism; V/Q, ventilation-perfusion.

**Table 2:** Costs (2013 US$) associated with diagnosis and treatment of venous thromboembolism and treatment-related adverse events.

Model variables	2013 US$	Reference
Major bleed	10,717	Deitelzweig et al, 2008 [16]
Minor bleed	5466	Deitelzweig et al, 2008 [16]
Asymptomatic HIT	1064	McGarry et al, 200 4[17]
Symptomatic HIT	14,032	McGarry et al, 2004 [17]
Treated deep vein thrombosis	10,758	Deitelzweig et al, 2008 [16]
Treated pulmonary embolism	19,032	Deitelzweig et al, 2008 [16]
Deep vein thrombosis diagnosis	449	Deitelzweig et al, 2008 [16]
Pulmonary embolism diagnosis	582	Deitelzweig et al, 2008 [16]
7 days of prophylaxis: enoxaprin (40 mg qd)*	380	Deitelzweig et al, 2008 [16]
7 days of prophylaxis: UFH (5000 IU bid)*	236	Deitelzweig et al, 2008 [16]

*Including $16 in pharmacy and nursing costs assumed per administration.

Bid, twice daily; HIT, heparin-induced thrombocytopenia; IU, international units; qd, once daily; UFH, unfractionated heparin.

The starting point of the decision-tree model was admission to an acute-care hospital due to a serious medical condition. If patients received LMWH or UFH, the drug-related adverse events (minor/major bleeds and symptomatic/asymptomatic HIT) were considered. Major bleeds and symptomatic HIT may lead to death. If patients survived drug-related adverse events, for simplicity, we assumed that they were not at risk of additional adverse events or VTE. Conversely, patients who did not experience HIT or bleeds were considered to be at risk of VTE. Although asymptomatic (venographically confirmed) DVT and fatal PE are generally accepted to represent a continuum, with a common pathological cause [52, 53], we considered the probability of true positive and false negative clinical diagnosis of DVT based on estimates derived from studies examining the prevalence of DVT in asymptomatic patients [39, 40]. Patients with a positive clinical diagnosis (confirmed on ultrasound, computed tomography or ventilation-perfusion scan) were presumed to be referred for VTE treatment. Different probabilities of getting PE after an episode of DVT with/without treatment were used in our decision-tree model. Patients with PE who survived the critical period immediately after the acute event (no sudden death) were divided into diagnosed or undiagnosed groups. Different PE-attributed case fatality rates were used based on different clinical scenarios (sudden death, with treatment, without treatment). The risk of death due to either drug-related adverse events or the underlying medical condition was taken as an endpoint in our decision-tree model as well (S1 Fig.).

#### Estimates of Effectiveness

Estimates of effectiveness, including those related to efficacy in preventing VTE or to drug-related adverse events, were based on the published literature (Table 1). The Prophylaxis in Medical Patients with Enoxaparin (MEDENOX) study [19] was used to estimate the risk of VTE with enoxaparin prophylaxis and the baseline risk of VTE among untreated patients. The results of a meta-analysis by Mismetti et al [18] were used to estimate the risk of prophylaxis failure among patients receiving UFH.

#### Cost Estimates

The economic evaluation of specific ACCP-recommended prophylaxis strategies (LMWH or UFH) relative to no prophylaxis from the US healthcare system perspective was undertaken using publicly available data, which included US wholesale prices for medication costs, and Medicare reimbursement rates by diagnosis-related group for treatment costs, as well as costs associated with the diagnosis and treatment of adverse events (Table 2).

Costs were analyzed based on direct medical costs from a US healthcare system perspective and were adjusted to 2013 US$ by using the medical-care component of the US Consumer Price Index [54]. Because the time horizon in this analysis is short, discounting was not required.

### Statistical Analysis

The primary outcome measure was the number of VTE-attributable deaths averted. Incremental costs, VTE-attributable deaths averted, and ICERs were calculated by comparison with no prophylaxis. In addition, we included the estimated costs and number of deaths averted associated with each 1% improvement in adherence to ACCP prophylaxis among medical inpatients at ACCP-defined VTE risk in the US acute care hospital setting. First, we estimated the cost and effectiveness for each type of ACCP-recommended prophylaxis provided to medical inpatients at VTE risk compared with no prophylaxis. Second, we applied this result to the entire US medical inpatient population at ACCP-defined VTE risk to estimate the US-wide implications of the primary outcome measure.

Considering the uncertainty of the probability of developing PE after an episode of DVT without treatment and the case-fatality rates among patients with PE that could impact on clinical outcomes, we conducted one-way sensitivity analyses to determine the threshold value of these estimates when the prophylaxis strategies begin to be effective. In addition, to estimate the total impact of the uncertainty of all parameters on our decision-tree model, we conducted probabilistic sensitivity analyses using Monte Carlo simulations (10,000 iterations) with β distributions for binomial variables and γ distribution for cost data.[55] Furthermore, we tested the robustness of the results by using the interquartile range (IQR) rates of medical inpatients at ACCP-defined VTE risk from each of the US sites in the ENDORSE study based on the base-case scenario of the decision-tree model.

We used SAS version 9.2 (SAS Institute Inc., Cary, NC, USA) to analyze ENDORSE and NIS data. We used TreeAge Pro 2009 decision analysis software (TreeAge Software, Williamstown, MA, USA) to perform the cost-effectiveness analyses.

## Results

Of the 68,183 inpatients enrolled in ENDORSE, 9257 were cared for in 81 US hospitals [11]. Of these patients, 5196 (56.1%) were cared for in medical wards, 2720 (52.3%) and were at ACCP-defined VTE risk, 1292 (47.5%) of whom received ACCP-recommended prophylaxis [11]. Clinically recognized contraindications to anticoagulant prophylaxis were present in 331 (12.2%) patients at ACCP-defined VTE risk. The proportion of medical inpatients at ACCP-defined VTE risk for each US hospital ranged from 19.2% to 100% (IQR: 44.8–62.2%).

**Fig 1:**
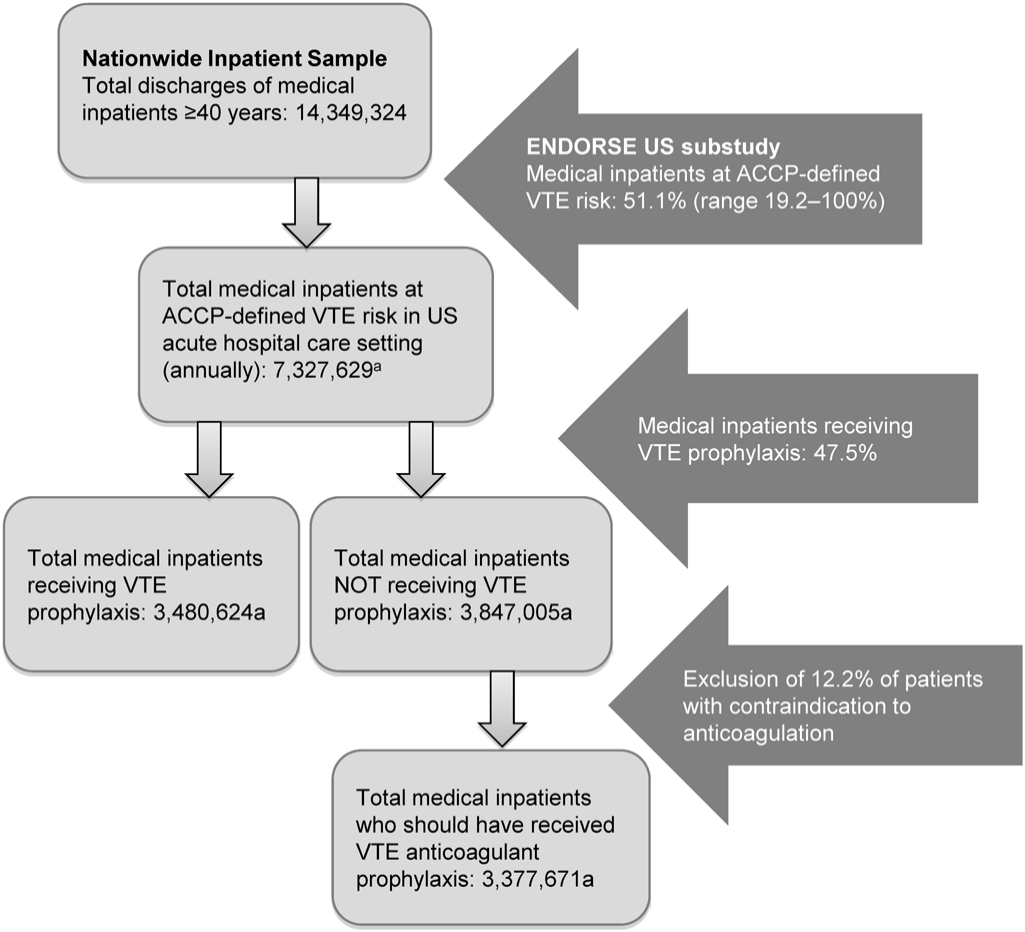
Application of findings from the ENDORSE US population [11, 15] to patients in the 2006 NIS population [13]. ^a^Calculated.

Based on year 2006 NIS data, 14.3 million medical inpatients aged ≥40 years and discharged from US acute care hospitals would have met the ENDORSE inclusion criteria. By extrapolating the ENDORSE findings to this NIS population, we estimated that 7.3 million medical patients aged ≥40 years would be at ACCP-defined VTE risk in the US acute care setting each year. Of these, an estimated 3.5 million patients would receive ACCP-recommended VTE prophylaxis and 3.8 million would not (Fig. 1) [11, 13, 15]. After excluding 12.2% of patients estimated to have clinically recognized contraindications to anticoagulants, 3.4 million medical patients should have received ACCP-recommended anticoagulant prophylaxis.

The results of our cost-effectiveness analysis, including the results from the probabilistic sensitivity analyses using Monte Carlo simulations, are shown in Table 3. Compared with no prophylaxis, the LMWH and UFH strategies would be expected to reduce the rates of deaths by 0.5% and 0.3%, respectively, translating into savings of $50,637 and $25,714 per death averted in base-case scenario. Applied to our base-case scenario estimate that on an annual basis there are 3.4 million eligible but unprotected medical patients in US acute care hospitals, thromboprophylaxis with LMWH or UFH would therefore be estimated to prevent 15,875 or 10,201 deaths annually, with total anticipated savings of $8 million for LMWH and $2.6 million, for UFH. Therefore, among medical inpatients at VTE risk who did not receive ACCP-recommended anticoagulation prophylaxis, each 1% increase in the use of LMWH prophylaxis would avert 159 deaths and save approximately $8 million dollars each year (Table 4). Alternatively, assuming that UFH was used, each 1% increase in prophylaxis would avert 102 deaths and save approximately over $2.6 million dollars each year.

**Table 3:** Cost-effectiveness analysis (2013 US$ among a hypothetical cohort of 10,000 acutely ill medical inpatients at ACCP-defined VTE risk).

Strategy	Total cost	Deaths	Incremental cost*	Death averted	Cost/death averted ($)*
Base-case					
No prophylaxis	$13,689,498	1087	–	–	–
Enoxaparin 40 mg qd	$11,309,543	1040	–$2,379,956	47	–$50,637
UFH 5000 IU bid	$12,918,092	1057	–$771,407	30	–$25,714
Low (2.5%) cases generated from Monte Carlo simulation (10,000 iterations)^§^					
No prophylaxis	$2,774,058	589	–	–	–
Enoxaparin 40 mg qd	$3,282,762	540	$508,704	49	$10,382
UFH 5000 IU bid	$4,312,000	555	$1,537,792	34	$45,234
High (97.5%) cases generated from Monte Carlo simulation (10,000 iterations)^§^					
No prophylaxis	$50,658,893	1762	–	–	–
Enoxaparin 40 mg qd	$28,887,687	1707	–$21,771,206	55	–$395,840
UFH 5000 IU bid	$32,954,385	1722	–$17,704,508	40	–$442,613

*A negative value indicates cost saved.

^§^ 95% of iterations fall between the low- and high-range.

ACCP, American College of Chest Physicians; bid, twice daily; IU, international units; qd, once daily; UFH, unfractionated heparin; VTE, venous thromboembolism.

**Table 4:** Estimated rates and numbers of deaths averted and associated cost savings, with adherence to VTE prophylaxis among medical inpatients at VTE risk in US acute care hospitals.

	LMWH (Enoxaparin 40 mg qd) vs. No Prophylaxis	UFH (5000 IU bid) vs. No Prophylaxis
Rate of deaths averted	0.5%	0.3%
Total deaths averted among medical inpatients at ACCP-defined VTE risk NOT receiving ACCP-recommended VTE prophylaxis	15,875	10,201
Cost saving per death averted	$50,637	$25,714
Total cost saving to prevent all deaths	$803,870,748	$262,292,916
VTE-attributable deaths averted for every 1% improvement in adherence to VTE prophylaxis	159	102
Total cost saving for every 1% improvement in adherence to VTE prophylaxis	$8,038,707	$2,622,929

ACCP, American College of Chest Physicians; bid, twice daily; ICER, incremental cost-effectiveness ratio; IU, international units; LMWH, low-molecular-weight heparin; qd, once daily; UFH, unfractionated heparin; VTE, venous thromboembolism.

**Fig 2:**
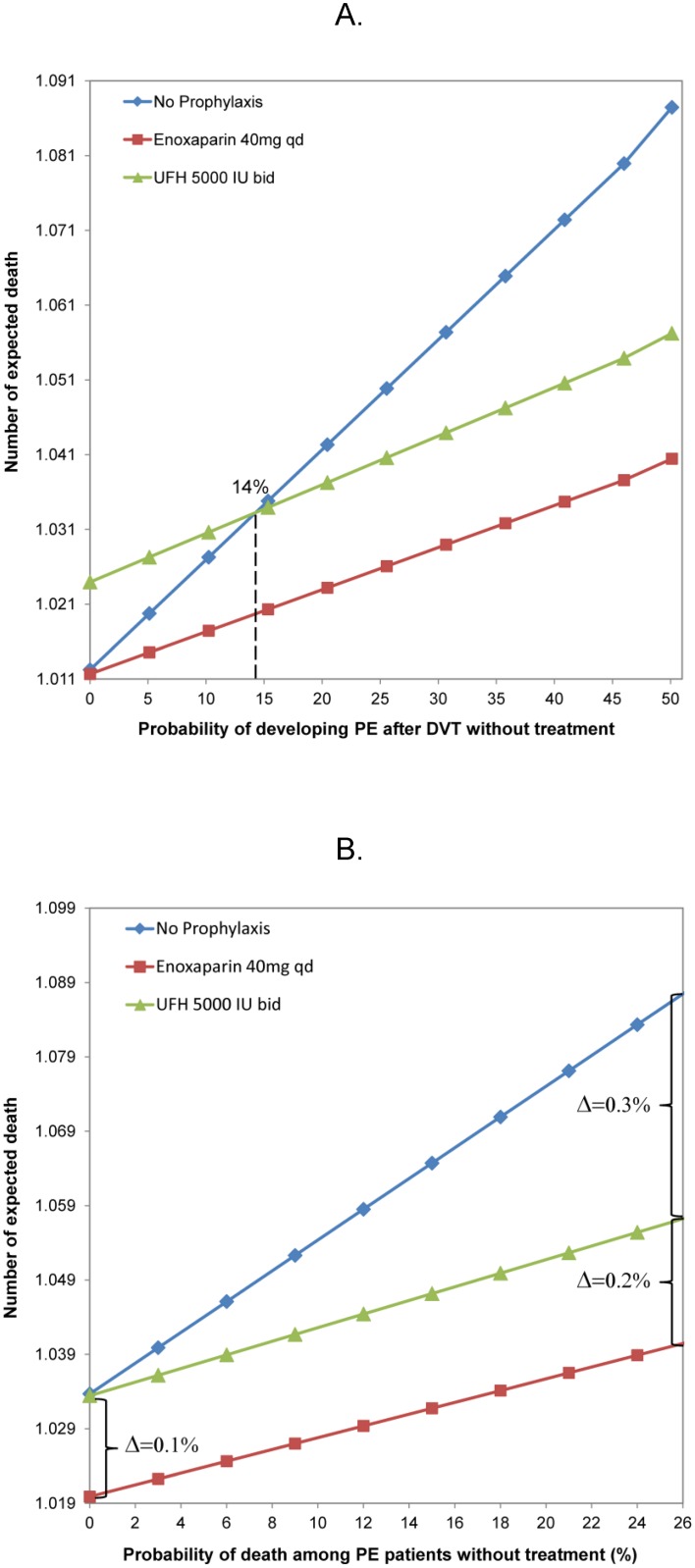
One-way sensitivity analyses to determine the threshold value of (A) the probability of PE after DVT without treatment; and (B) probability of death among PE patients who survived the period immediately after the acute event without treatment, among 10,000 acutely ill medical inpatients at ACCP-defined VTE risk. ACCP, American College of Chest Physicians; bid, twice daily; DVT, deep vein thrombosis; IU, international units; PE, pulmonary embolism; qd, daily; VTE, venous thromboembolism.

**Fig 3:**
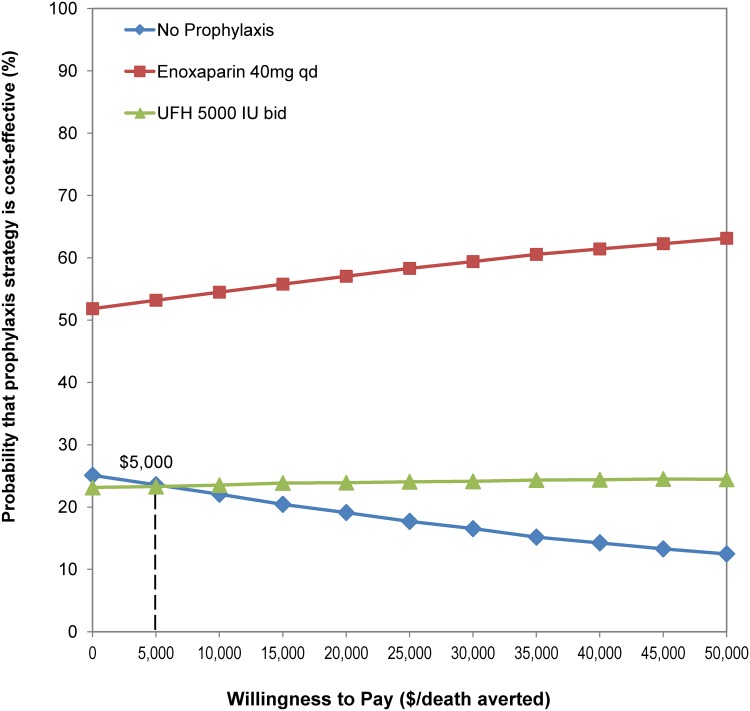
Cost-effectiveness acceptability curve based on a Monte Carlo simulation (10,000 iterations). bid, twice daily; IU, international units; qd, daily; VTE, venous thromboembolism.

One-way sensitivity analyses of key estimates in our decision-tree model indicated that as long as the probability of developing PE after DVT without treatment is above 14%, both prophylaxis strategies are cost-effective compared with no prophylaxis (Fig. 2A). Second, the probability of death among PE patients who survived from the critical period immediately after the acute event increased from 0% to 26% (point-estimate in the decision-tree model); the overall reduction in death rates ranged from 0.1% to 0.5% for the LMWH strategy, and from 0% to 0.3% for the UFH strategy, compared with no prophylaxis (Fig. 2B). Third, the LMWH strategy is always dominant (Fig. 2A, B), regardless of the exact value of the two estimates. The Monte Carlo simulation with 10,000 iterations indicated that: (1) regardless of the cost per death averted, over 50% of iterations indicated that the LMWH strategy is cost-effective; (2) if the healthcare system is willing to pay approximately $5,000 per death averted, the UFH strategy starts to have higher probability of being cost-effective compared with no prophylaxis (Fig. 3).

The annual proportion of medical inpatients at ACCP-defined VTE risk in the US acute care setting varies among the 81 US ENDORSE hospitals (IQR: 44.8–62.2%). Applying this variation to the base-case of the decision-tree model, the range of deaths averted each year would range from 13,927 to 19,336 for a LMWH strategy and from 8949 to 12,425 for a UFH strategy; these figures translate to annual total costs saved of between $705 to $979 million for LMWH, and $230 to $319 million for UFH. For each 1% increase in ACCP-recommended prophylaxis applied to at-risk medical inpatients, the range of annual deaths averted would be 139 to 193 for the LMWH strategy and 89 to 124 for the UFH strategy, with a total estimated savings of $7 to $9.8 million and $2.3 to $3.2 million, respectively.

## Discussion

While a number of studies have suggested that providing anticoagulant prophylaxis to medical inpatients is highly cost-effective compared with no prophylaxis [16, 17, 56], the present paper provides new estimates of the magnitude of the opportunity to prevent VTE outcomes including attributable deaths in US acute care hospitals by providing prophylaxis to hospitalized medical patients at VTE risk, with costs estimated from a US healthcare system perspective. Moreover, by providing an objective assessment of the proportion of at-risk patients who have a known contraindication to anticoagulant prophylaxis, we provide an estimate of the system-wide impact on both deaths and costs, assuming US hospitals fully implement the revised 2012 ACCP recommendations.

### Cost-effectiveness of Thromboprophylaxis in Hospitalized Medical Inpatients

McGarry et al, in 2004, estimated that the incremental cost in 2001 USD per death averted with LMWH prophylaxis versus no prophylaxis in acutely ill medical inpatients was $9100, and LMWH prophylaxis dominated UFH prophylaxis [17]. By contrast, in the present analysis, although our result on the effectiveness of anticoagulant prophylaxis was similar to that reported in the McGarry study, we estimated that provision of VTE prophylaxis to a similar population would produce cost savings in 2013 USD per death averted of over $50K for LMWH and $25K for UFH. The key difference between the McGarry et al data and our analyses is driven by the cost of US hospital inpatient services, which have increased substantially since 2001 [57]. These included increases in costs related to both the diagnosis of VTE and the treatment of VTE and of adverse events. In particular, the cost related to treatment of DVT increased from $3245 to $10,758, and the cost related to PE treatment increased from $8367 to $19,032, even after adjusting 2001 US$ to 2013 US$ using the medical-care component of the US Consumer Price Index [9, 16, 17]. In the present analysis we estimate that provision of VTE prophylaxis for hospitalized medical patients not only saves lives but also saves dollars from a healthcare-systems perspective, by avoiding the expenditure of precious resources on the diagnosis and treatment of preventable episodes of VTE.

Our findings are similar to a number of other published studies that examined the clinical and cost-effectiveness of thromboprophylaxis with LMWH and UFH versus placebo in medical patients [16, 56]. In a paper published in 2008, Deitelzweig et al modeled 2-year outcomes and costs of prophylaxis in medical inpatients at risk of VTE, finding that an average medical cost per patient within 30 days post admission could be reduced by 2006 US$347 by using LMWH prophylaxis, compared with no prophylaxis, and by 2006 US$89 by using UFH prophylaxis [16]; both LMWH and UFH prophylaxis would lead to additional savings when this analysis was extended through 2 years post admission [16]. Extension of this time window beyond 30 days would likely lead to a further reduction in deaths from VTE and to greater cost savings. Although our analyses provide an estimate of the range of clinical and economic impacts of improving ACCP-recommended VTE prophylaxis in the US hospital care setting annually, the full impact of the current missed opportunities to provide prophylaxis to at-risk medical patients may be underestimated. In particular, owing to the aging of the population, increasing numbers of older patients require hospitalization for a wide variety of acute medical illnesses. Thus the number of candidates for VTE prevention is rising and may be substantially higher than previously estimated [58], and the clinical and economic impact of every 1% increase in adherence to ACCP-recommended VTE prophylaxis may exceed our estimates.

We acknowledge that the uncertainty of the parameter estimates used in the decision-tree model could influence the study outcome. In particular, the rates of probability of VTE for each strategy, the probability of developing PE after DVT, and the death rates associated with PE could have an important effect. The results of our sensitivity analyses and Monte Carlo simulation indicate that LMWH prophylaxis is a dominant strategy in our model, primarily due to a lower rate of drug-associated adverse events compared with the UFH strategy and a substantially lower rate of VTE compared with the no-prophylaxis strategy. When comparing UFH prophylaxis with no prophylaxis, the threshold value of the PE rate after DVT without treatment was 14%, to be cost-effective, based on the one-way sensitivity analysis. This is much lower than the estimates reported in other studies [36, 37]. Furthermore, paying US$5000 per death averted, as illustrated in the acceptability curve based on Monte Carlo simulation, is well below the often-cited threshold of US$50,000 per quality-adjusted life-year (QALY) gained (assuming each death averted results in a gain of just one QALY) [17]. Lowering the PE-associated mortality rate would not change the direction of our conclusion, as these changes in absolute rates would occur in the same direction for each of the strategies compared.

### Implications of the Revised 2012 ACCP Recommendations

The 2004 ACCP recommendations, on which these analyses are based, advocate that hospitals develop standing orders for VTE prophylaxis in broad classes of patients hospitalized with major medical illness. The recently revised (2012) ACCP guidelines recommend that physicians take an individualized approach to provision of VTE prophylaxis, balancing the benefits of VTE prevention against bleeding risks in each patient. Comparing 2004 and 2012, there are no significant differences in the groups of medical patients at risk of VTE that are recommended to receive thromboprophylaxis; however, there are small differences with respect to the risk of bleeding. Based on the ENDORSE findings in US hospitals, approximately 12% of patients hospitalized with a severe medical illness have a recognized risk of bleeding sufficient to preclude the use of anticoagulant prophylaxis. Therefore the potential impact of the 2012 ACCP recommendations-namely that physicians withhold anticoagulant prophylaxis from patients at known high risk for bleeding-would at most reduce the number of patients who should receive VTE prophylaxis in the US each year by approximately 12%.

Additional research is needed to estimate the costs and the unknown benefits of mechanical methods of thromboprophylaxis in the subset of patients who are hospitalized for treatment of major medical conditions but who cannot receive anticoagulant prophylaxis due to a recognized high risk for bleeding.

### Study Limitations

This study was based on robust US-wide data from the NIS and ENDORSE. Those 81 US ENDORSE hospitals were randomly selected from the American Hospital Association database. The same patient-selection algorithm was used in both NIS and ENDORSE. Consequently, the findings from the present analysis are highly likely to be generalizable to patients cared for in US hospitals. Despite the strengths, we recognize a number of potential limitations, including: uncertainties in our estimates of rates and costs; inclusion of a heterogeneous group of patients with severe medical illnesses who have a high, but variable, risk of VTE; not accounting for the longer term consequences of VTE; changes in practice between 2006 and 2013 in terms of improved uptake of current therapies, if any; the extent of adoption of newer prophylactic agents; and changes in healthcare costs for prevention, diagnosis and treatment of VTE and its associated complications. However, these cost estimates do provide a benchmark for this setting. In addition, our decision-tree model is necessarily a simplified representation of VTE and its management, and does not include all possible strategies and outcomes. Also, the use of US wholesale prices for medication costs and Medicare reimbursement rates by diagnosis-related group for treatment costs may not represent the real costs that specific institutions or payers incur. Individual institutions may negotiate a different purchase price for each medication, and specific payers may have a different reimbursement rates for each treatment compared with the payment rates we employed in our model to estimate hospital costs. The introduction of new classes of treatment, such as direct thrombin inhibitors and factor Xa inhibitors, presents patients, clinicians and the healthcare system with additional therapeutic choices; however, none are approved or likely to be approved in the next few years. In addition, many patients hospitalized for severe medical illnesses will have a limited life expectancy due to the presence of other, terminal conditions, especially cancer. Further research is needed to quantify the number of months that VTE prophylaxis delays death in patients who have a poor prognosis, irrespective of fatal PE, which is a potentially preventable condition.

## Supporting Information

S1 Fig Decision-tree model based on model published by McGarry et al [17], with updated cost estimates. (PDF)
