# Supplementary material for: Impact of Thromboprophylaxis across the US Acute Care Setting
Source: PLoS One. 2015 Mar 27;10(3):e0121429. doi: 10.1371/journal.pone.0121429 (PMC4376674; doi:10.1371/journal.pone.0121429)
Supplement: S1 Fig — (PDF) [file pone.0121429.s001.pdf]

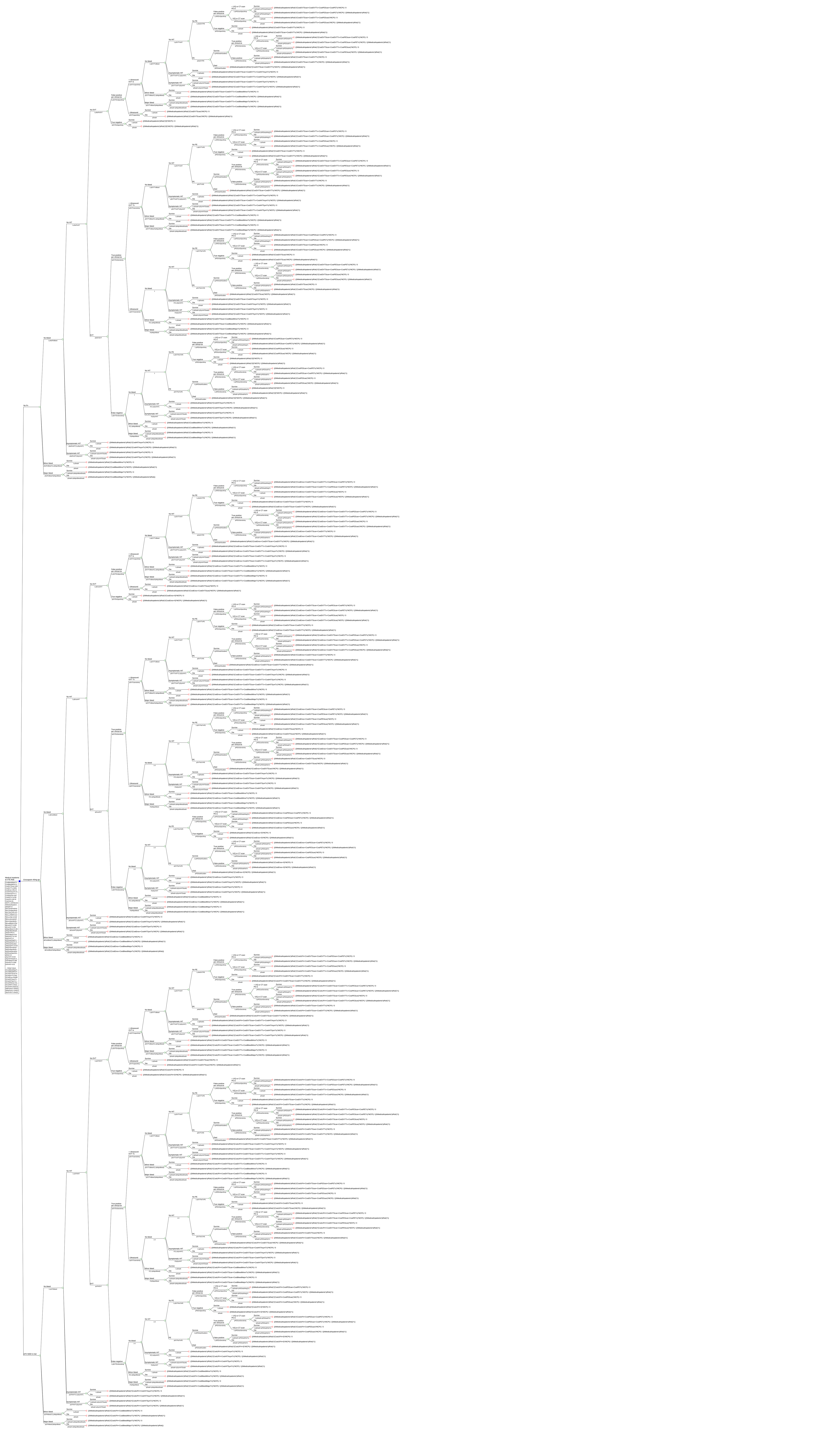
Medical Inpatients at VTE Risk
Enoxaparin 40mg qd
CostBleedMajorTx...
CostBleedMinorTx...
CostDVTScan=349.
CostDVTTx=8355.
CostEnox=295.19
CostHITAsymTx=8...
CostHITSymTx=1...
CostPEScan=452.
CostPETx=14781.
CostUFH=182.91
CostVisit=61.
MCPI=1.287591422
NMedicalInpatient...
pDeath=0.1
pDVTDxSensitivity...
pDVTDxSpecificity...
pDVTNoTxPE=0.5...
pDVTTxBleed=0.0...
pDVTTxHIT=0.012
pDVTTxPE=0.018
pDVTUsensitivity=...
pDVTUspecificity=...
pEnoxBleed=0.031
pEnoxDVT=0.055
pEnoxHIT=0.001
pMajorBleed=0.185
pMajorBleedDeath...
pNoDVTPE=0.
pNoPxBleed=0.02
pNoPxDVT=0.142
pNoPxHIT=0.
pPEDeathNegTx=...
pPEDeathNoTx=0....
pPEDeathSudden...
pPEDeathTx=0.015
pPEDxSensitivity=...
pPEDxSpecificity=...
pPEScanSensitivit...
pPEScanSpecificit...
pRisk=0.5
pSymHIT=0.543
pSymHITDeath=0....
pUFHbleed=0.058
pUFHDVT=0.066
pUFHHIT=0.01
--- Global Values
DCostBleedMajorT...
DCostBleedMinorT...
DCostDVTScan=D...
DCostDVTTx=Dist...
DCostEnox=Dist(8)
DCostHITAsymTx...
DCostHITSymTx=...
DCostPEScan=Dis...
DCostPETx=Dist(1...
DCostUFH=Dist(13)
DpEnoxDVT=Dist(2)
DpNoPxDVT=Dist(1)
DpUFHDVT=Dist(3)
